# Comparative Analysis of Active LTR Retrotransposons in Sunflower (*Helianthus annuus* L.): From Extrachromosomal Circular DNA Detection to Protein Structure Prediction

**DOI:** 10.3390/ijms252413615

**Published:** 2024-12-19

**Authors:** Mikhail Kazancev, Pavel Merkulov, Kirill Tiurin, Yakov Demurin, Alexander Soloviev, Ilya Kirov

**Affiliations:** 1 All-Russia Research Institute of Agricultural Biotechnology, Timiryazevskaya Str. 42, 127550 Moscow, Russia; irelanddets@gmail.com (M.K.); paulmerkulov97@gmail.com (P.M.); tiurin.kn@gmail.com (K.T.); a.soloviev70@gmail.com (A.S.); 2 Pustovoit All-Russia Research Institute of Oilseed Crops, Filatova St. 17, 350038 Krasnodar, Russia; genetic@vniimk.ru; 3 All-Russia Center for Plant Quarantine, 140150 Ramenski, Russia

**Keywords:** additional ORF, *Tekay*, Chromovirus, eccDNA, nanopore sequencing, protein 3D structure

## Abstract

Plant genomes possess numerous transposable element (TE) insertions that have occurred during evolution. Most TEs are silenced or diverged; therefore, they lose their ability to encode proteins and are transposed in the genome. Knowledge of active plant TEs and TE-encoded proteins essential for transposition and evasion of plant cell transposon silencing mechanisms remains limited. This study investigated active long terminal repeat (LTR) retrotransposons (RTEs) in sunflowers (*Helianthus annuus*), revealing heterogeneous and phylogenetically distinct RTEs triggered by epigenetic changes and heat stress. Many of these RTEs belong to three distinct groups within the *Tekay* clade, showing significant variations in chromosomal insertion distribution. Through protein analysis of these active RTEs, it was found that *Athila* RTEs and *Tekay* group 2 elements possess additional open reading frames (aORFs). The aORF-encoded proteins feature a transposase domain, a transmembrane domain, and nuclear localization signals. The aORF proteins of the *Tekay* subgroup exhibited remarkable conservation among over 500 *Tekay* members, suggesting their functional importance in RTE mobility. The predicted 3D structure of the sunflower *Tekay* aORF protein showed significant homology with *Tekay* proteins in rice, maize, and sorghum. Additionally, the structural features of aORF proteins resemble those of plant DRBM-containing proteins, suggesting their potential role in RNA-silencing modulation. These findings offer insights into the diversity and activity of sunflower RTEs, emphasizing the conservation and structural characteristics of aORF-encoded proteins.

## 1. Introduction

LTR retrotransposons (RTEs) are a major component of most plant genomes. In addition to being the major structural element of the genome, RTEs also produce RNA and proteins during their lifecycle. Autonomous RTEs usually express six proteins, including two group-specific antigen (GAG)-derived proteins (capsid (CA) and nucleocapsid (NC)) and five polyprotein (POL)-derived proteins (aspartic proteinase (AP), reverse transcriptase (RT), RNase H (RH), and integrase (INT)) [1]. These proteins have long been known as core proteins required for the RTE lifecycle [2]. During their lifecycle, LTR retrotransposons are transcribed by host polymerase II in the nucleus, and the resulting RNA is translated into GAG and POL proteins in the cytoplasm [3]. In virus-like particles formed by GAG in the cytoplasm, reverse transcription of the retrotransposon genomic RNA is mediated by retrotransposon RT and RNase H, formed during pol processing by AP. Integration of the resulting cDNA is mediated by a protein dimer formed by INT, which, particularly in Chromoviruses, may include a chromodomain (CHD) that promotes its localization in heterochromatin [4]. In this way, canonical proteins not only determine the completeness of the replication cycle but are also implicated in integration site selection. According to the ‘tethering model’, RTEs can have insertion preferences caused by interactions between the RTE integration complex and chromatin proteins [5]. The earliest example of this is the prevalent integration of yeast *Ty3*/*Gypsy* into PolIII-transcribed genes caused by the interaction between the integration complex and PolIII transcription factors (TF) III-B and -C [6]. Integration preferences have also been observed for some plant RTEs, such as *Ty1*/*Copia ONSEN* elements, which preferentially integrate into H2A.Z-enriched chromatine [7]. Moreover, redistribution of H2A.Z toward the pericentromeric region in the ddm1 (decreased DNA methylation 1) mutant leads to corresponding changes in *ONSEN* integration site density along the chromosome [8]. 

While canonical proteins are implicated in the RTE replication cycle, some RTEs can also encode extra proteins from additional open reading frames (aORFs) [9,10]. One of the first examples of these additional proteins is the highly divergent group of envelope-like (env-like) proteins [11,12,13]. Although these proteins are involved in the intercellular transfer of retroviruses, their functional role in the RTE replication cycle is debatable [1,14]. Interestingly, RTEs may have additional open reading frames (aORFs) without any structural similarity to known retroviral or plant proteins [9]. Such aORFs have been described for different plant species, including ORF23 of *Grande*1 element of *Zea* species [15,16], orf3 of *RIRE*3 element of *Oryza sativa* [17,18], ORF3/4 of *Retand*-2 element of *Silene latifolia* [19], multiple aORFs of *Tat*/*Athila* and Chromovirus lineages of *Arabidopsis thaliana* [10], and others. While aORFs’ classification has not been established, they are usually divided into two groups, 5′aORFs (e.g., Chromovirus elements) and 3′aORFs (e.g., *Athila* elements), according to their localization with respect to the GAG-POL ORF [10]. Occasionally, RTEs may have both types of aORFs [10,20]. Although some aORFs are similar to certain plant protein domains [10], the evolutionary origin and functions of aORF proteins or peptides remain unknown. 

Sunflower (*Helianthus annuus* L.) is one of the world’s four major annual crops grown for food oils. This oil is rich in unsaturated fatty acids, contains high amounts of vitamin E, and is easily refined [21]. The main place of origin is North America, from where it spread all over the world [22,23], but sunflower has become an oilseed crop with high oil content (47–51% of oil in seeds) in Russia because of studies in the field of mutagenesis by V.S. Pustovoit and his followers [24]. The sunflower genome contains a bulk of repetitive elements, with approximately 75% of the genome occupied by RTEs. RTEs of the *Ty*3/*Gypsy* superfamily are more abundant than *Ty1*/*Copia* elements in the sunflower genome [25]. We and others have shown that tens of these RTEs are transcriptionally active, with *Ty1*/*Copia* (*Ale* family) RTEs significantly overrepresented among the expressed RTEs [26,27,28,29]. A comparison of the copy numbers of expressed and non-expressed RTEs showed that the former are often low-copy elements, whereas the second group includes multi-copy RTEs, such as *Tekay* [27]. The expressed RTEs of sunflowers have been invoked in transposition activity analysis performed by PCR with specific primers and genomic DNA of different varieties [29]. More recently, the transposition activity of expressed sunflower RTEs was elucidated by PCR analysis of the extrachromosomal circular DNA (eccDNA).

EccDNAs are generated from the cDNA of RTEs during the final stage of their lifecycle [30]. Therefore, eccDNAs have been used as proxies for RTE transposition activity in plants [31]. The PCR assay of eccDNAs for sunflower RTEs revealed expressed and transpositionally active RTEs, such as *Gagarin* and *SUNTY*3 [26,27]. The overall composition of eccDNAs in plants can be determined by next-generation sequencing using the Mobilome-Seq method [32]. This approach has been used to elucidate transposition activity and identify RTEs in different plant species [31,33,34]. However, the short length of Mobilome-Seq reads makes it challenging to precisely identify active RTE copies in plants with large genomes, such as sunflowers. Recently, eccDNA sequencing using long-read technologies (PacBio and Oxford Nanopore) was conducted for *A. thaliana* [35,36], *T. aestivum* [37], and *S. lycopersicum* [38]. The long-read version of Mobilome-Seq (ONT Mobilome-Seq) is a promising approach for genome-wide identification of active RTEs in plants with complex genomes.

In this study, we aimed to survey transpositionally active sunflower RTEs at the whole-genome level. Using ONT Mobilome-Seq, we identified 28 RTEs of different lineages of *Ty3*/*Gypsy* and *Ty1*/*Copia* that produced eccDNAs under provocative stress conditions. We identified three phylogenetic clusters of *Tekay* RTEs with contrasting insertion distribution patterns along the chromosome, and found non-canonical proteins encoded by additional ORFs. Overall, the present study provides a list of active sunflower LTR retrotransposons and shows that these RTEs encode a subset of structurally distinct non-canonical proteins of unknown functions.

## 2. Results

### 2.1. Identification of Active Sunflower LTR Retrotransposons by Nanopore Sequencing of Extrachromosomal Circular DNA (eccDNA)

**Figure 1:**
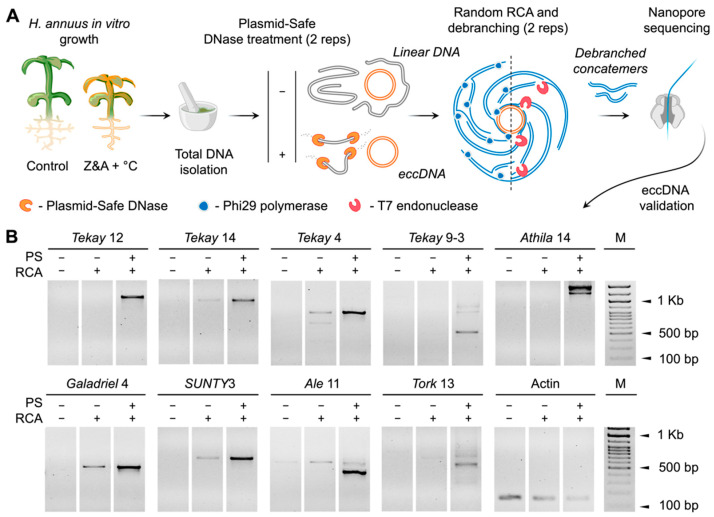
(**A**) Schematic overview of the sunflower mobilome activation and eccDNA sequencing experiment. (**B**) Validation of the detected eccDNA by inverted PCR with specific primers.

To investigate the features of transpositionally active sunflower RTEs, we conducted genome-wide analysis of RTE activity. To this end, we triggered RTE activity by applying heat stress in combination with ‘epigenetic stress’, which involves growing plants on a medium containing two toxins, zebularine (Z) and α-amanitin (A), as previously described [26] (Figure 1A). To identify the active RTEs, we used nanopore sequencing of eccDNA (ONT Mobilome-Seq). To increase the sensitivity of ONT Mobilome-Seq, we divided the total DNA into two parts: with (PS+) and without (PS−) treatment with Plasmid-Safe DNase (PS). Both variants underwent rolling circle amplification (RCA; Figure 1A). The RCA products were purified, either treated with T7 endonuclease or proceeded without T7 treatment. T7 endonuclease treatment was used for RCA product debranching to improve nanopore sequencing. Nanopore sequencing yielded 502,000 and 452,000 reads for the PS− and PS+ samples, respectively. The number of reads obtained for T7-treated samples was significantly higher (376,484 and 380,826 reads per PS− and PS+ sample, respectively) than that in the samples without T7 treatment (125,720 and 71,652 reads per PS− and PS+ sample, respectively). These results showed that T7 treatment significantly increased the nanopore sequencing output of the RCA products. We also estimated the rate of eccDNA enrichment by analyzing the coverage of the homologous sequence of the P1 mitochondrial plasmid. P1 plasmids were present in some sunflower lines, as previously observed [39]. We found that the homologous sequence of P1 was located on chromosome 12 (position: 3,404,803…3,405,906) of the XRQ 2.0. We also found that the P1 locus had 20 times higher coverage in the PS+ sample (92,000×) than in the PS− sample (4000×), suggesting sufficient enrichment and reliability of the eccDNA isolation procedure used in this study. 

Next, we analyzed the eccDNAs originating from RTEs using a previously established protocol for ONT Mobilome-Seq data analysis [36] and nanopore reads from the PS+ and PS− samples. The Fisher’s exact test *p*-value of 0.01 was used as a cutoff for the comparison. Additionally, we manually curated the RTEs to select those uniformly covered by eccDNA reads. This analysis yielded 28 RTEs (Table S1). To validate these RTEs, we designed specific primers (Table S2) for inverted PCR (Supplementary Figure S1A). PCR was conducted for PS+, PS−, and control (total genomic DNA without RCA and PS treatment) samples. PCR with primers for a housekeeping gene (actin) was used as a control for linear DNA. For validation, we used only primers that did not produce PCR products with total DNA, without the eccDNA enrichment procedure (Supplementary Figure S1B). Primers for nine RTEs, including *SUNTY*3 [26], were selected (Figure 1B). Inverted PCR with these primer pairs resulted in stronger PCR products for the PS+ RCA samples than for the PS– and control RCA samples. In turn, PCR products with primers on actin showed the opposite pattern (a clear PCR band for control DNA and a weaker band for the PS+ sample). These results demonstrated the reliability of the proposed approach. 

Overall, we identified 28 active sunflower RTEs that produced eccDNA under stressful conditions.

### 2.2. Phylogenetic Analysis of the Active Sunflower RTEs

The identified active RTEs were classified using phylogenetic analysis, based on their RT and INT amino acid sequences. We found that the vast majority (22 elements) of the active RTEs belonged to the *Gypsy* superfamily, and only six RTEs were classified as *Copia* elements (Figure 1A). Further phylogenetic analysis of the RTEs using lineage- and clade-specific sequences of known RTEs revealed that the *Copia* elements included RTEs from two lineages: *Ale* (three RTEs) and *Tork* (three RTEs). *Gypsy* RTEs were represented by the following clades: *Athila* (1 RTE), *Galadriel* (4 RTE), and *Tekay* (17 RTE). This revealed that the most abundant clade in our analysis, *Tekay*, was divided into three phylogenetic groups (groups 1–3). The phylogenetic relationship between RTEs showed high sequence heterogeneity in each clade, except for group 1, which consisted of 10 highly similar RTEs.

**Figure 2:**
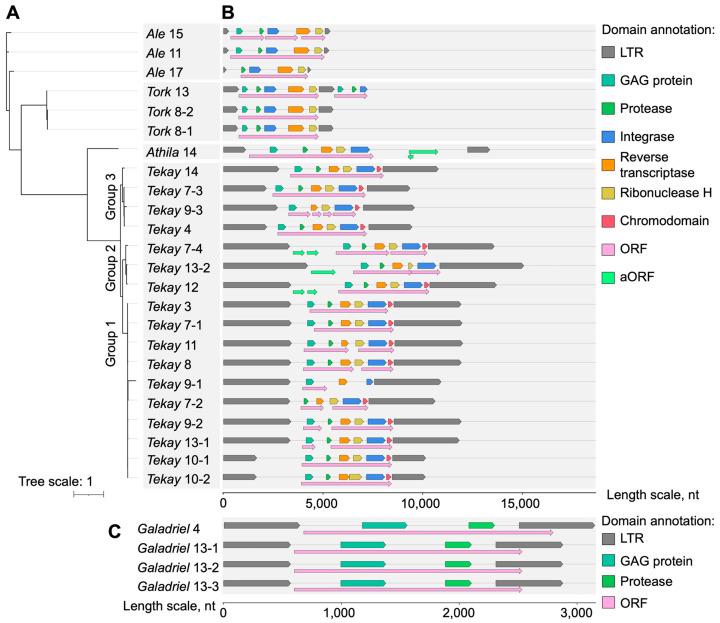
Phylogenetic and structural analyses of active sunflower RTEs. (**A**) Phylogenetic tree for 24 RTEs with detected activity (*Galadriel* elements excluded). (**B**) Annotation of predicted domains and open reading frames (ORFs) for RTEs used in phylogenetic analysis. (**C**) Domain annotation for TR-GAG *Galadriel* RTEs.

We analyzed the domain structures of all 28 RTEs. The majority of the RTEs in each clade contained all domains (20 full-domain elements in total), except for the *Galadriel* clade, where only the GAG domain was identified for all members (Figure 2C). These RTEs are likely TR-GAG elements of sunflowers, which were described at the transcriptome level in our previous work [26]. In addition, *Galadriel* 13-1, 13-2, and 13-3 are three nearly identical tandem organized elements with mutual LTRs located between the body sequences. The same organization was also detected for pairs of elements *Tork* 8-1 and 8-2, and *Tekay* 10-1 and 10-2. In other clades, there were only a few elements that were characterized by one or more domain losses (no GAG domain in *Ale* 17 and *Tekay* 7-2, no PROT in *Tekay* 9-3, and loss of PROT, RH, and CHD in *Tekay* 9-1). 

Altogether, these results showed that active sunflower RTEs are structurally heterogeneous and phylogenetically different, with the majority of RTEs belonging to three groups of the *Tekay* clade.

### 2.3. Insertional Landscape of Active RTEs in Sunflower Cultivars

**Figure 3:**
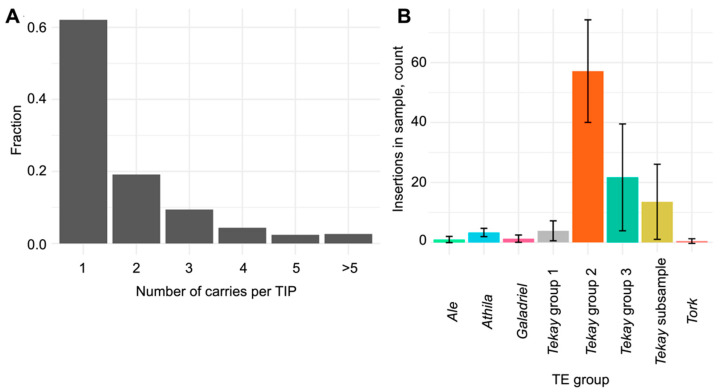
TIP analysis. (**A**) Fraction of TIPs shared by different numbers of sunflower genotypes. (**B**) Number of TIPs per TE group.

To investigate the insertional landscape of active RTEs, we analyzed the TE insertion polymorphism (TIP) using sequencing data from 12 sunflower cultivars using SPLITREADER (see Section 4). In total, we identified 2624 TIPs generated by 28 active RTEs. More than 80% of these were present in one or two accessions, suggesting that TIPs occurred during recent sunflower genotype diversification (Figure 3A) [40]. The majority of the identified TIPs originated from RTEs of the *Tekay* clade (group 2; Figure 3B).

**Figure 4:**
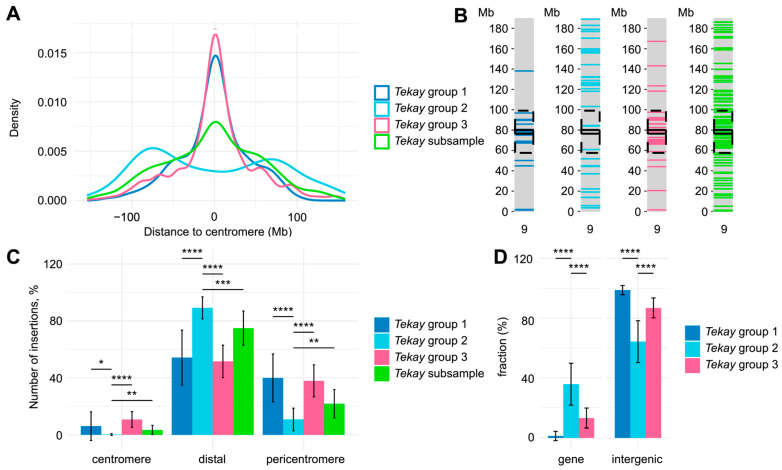
Chromosomal distribution of TIPs. (**A**) Density of TIPs in three *Tekay* groups and one group of randomly sampled *Tekay* RTEs across 17 sunflower chromosomes. (**B**) Physical distribution of *Tekay* TIPs on sunflower chromosome 9 as an example, where light green, light blue, and red indicate groups 1, 2, and 3, respectively. Pericentromeric and centromeric regions are marked by dotted and one-piece lines. (**C**) Distribution of TIPs across chromosome parts in 13 sunflower accessions. (**D**) Distribution of gene-related and intergenic insertions of *Tekay* TIPs in 13 sunflower accessions. Statistical significance is marked as ****, ***, **, and * for *p*-values lower than 0.0001, 0.001, 0.01, and 0.05, respectively, using the *t*-test.

The number of TIPs produced by the *Ale*, *Athila*, *Galadriel*, and *Tork* RTE clades was smaller (with maximum 3.3 ± 1.4 TIPs per sample) compared to the *Tekay* clade RTEs (Figure 3B), which accounted for 3.9 ± 3.3 to 57.2 ± 17.1 TIPs per sample. The three *Tekay* groups significantly differed in the number of detected TIPs, with group 2 exhibiting the highest value. We compared the chromosome distribution of RTEs of the three *Tekay* groups and found clear differences in the chromosome insertion distribution between groups 1/3 and 2. To assess the non-randomness of all *Tekay* group distribution patterns, we conducted TIP analysis with 16 randomly sampled *Tekay* elements (Supplementary Figure S2). We found that TIPs of *Tekay* groups 1 and 3 showed centromere-biased distribution patterns, whereas TIPs of group 2 were mostly located in the chromosome arms (Figure 4A–C; Supplementary Figures S3–S5). Next, we evaluated the fraction of TIPs located in the genes and found that TIPs of *Tekay* group 2 were frequently located in or near (+/−2000 bp nearby regions) the annotated protein-coding genes (Figure 4D; Supplementary Figure S6). 

Thus, the TIP survey provided evidence that the identified *Tekay* RTEs significantly differed in the number of TIPs per sunflower accession, as well as in TIP distribution along the chromosome, with RTEs of group 2 generating the highest number of TIPs that were predominantly distributed in the chromosome arms.

### 2.4. Comparative Analysis of Integrase and Chromodomain Sequences of Active Tekay RTEs

Given the euchromatic distribution of the novel insertions produced by the *Tekay* 2 group, in contrast to the pericentromere preference of groups 1 and 3, we performed a comparative analysis of the amino acid sequences of the integrases. Multiple alignments of integrases of the detected *Tekay* elements selected from each group by the highest number of TIPs showed the presence of a zinc finger with an HHCC motif at its *N*-terminus and a core domain containing the D and D(35)E motifs around the active site (Supplementary Figure S7A). We constructed 3D models of integrases and failed to identify any intragroup features of this domain (Supplementary Figure S7B). We then performed multiple alignments of the chromodomains of the three *Tekay* groups with known heterochromatin protein 1 (HP1) from *Drosophila melanogaster* and chromodomain II from previously described elements. We found that the chromodomains of *H. annuus Tekay* differed significantly from those of the other species. The similarity of chromodomains within *Tekay* groups 2 and 3 was >82% and >93%, respectively. Chromodomain similarity within group 1 varied from 44 to 97%. Similar to canonical chromodomain group 2, all sunflower *Tekay* chromodomains contained only one aromatic base (W at the 26 position of alignment), involved in aromatic pocket formation (Supplementary Figure S8). Intergroup comparisons of chromodomains revealed that groups 1 and 3 had more common residues, whereas group 2 chromodomains had more unique amino acid substitutions. Thus, although the *Tekay* elements of the three groups had chromodomain II sequences, the chromodomain of the group 2 elements formed a distinct phylogenetic cluster.

### 2.5. Structural Features’ Additional RTE Proteins

**Figure 5:**
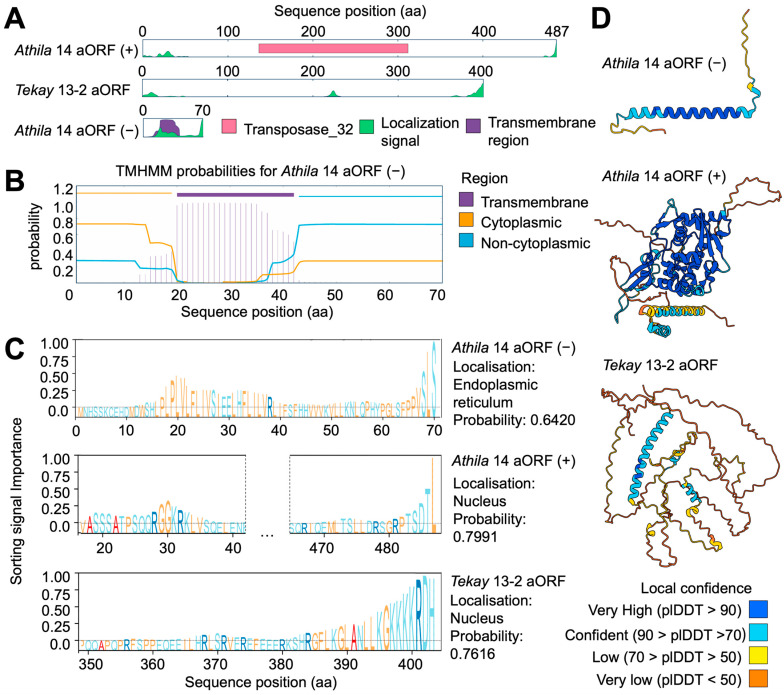
(**A**) Overview of aORF translation products. (**B**) Transmembrane signal prediction for *Athila* 14 aORF(−). (**C**) Localization signal prediction. (**D**) AlphaFold 3 predicted structures of additional aORF translation products.

To identify the putative proteins encoded by the identified active RTEs, we predicted all the ORFs (Figure 2B). We found that in addition to ORFs for GAG and POL, there were several ORFs that encoded proteins without similarity to known RTE proteins. These ORFs included the *Athila* 14 ORF(+) (487 aa), *Athila* 14 ORF(−) (70 aa), and *Tekay* 13-2 ORF(+) (402 aa). A search for domain similarity to other plant proteins in the PFAM database revealed partial similarity of the *Athila* 14 ORF(+) (positions 133-312) to Transposase_32 (PF20167), which is related to ORF1 of *Athila* elements in *A. thaliana* (PF03078; Figure 5A). The proteins of the *Athila* 14 ORF(−) and *Tekay* 13-2 ORF(+) were not similar to the known domains. We estimated the physicochemical properties of the predicted proteins to gain a deeper insight into the protein. Since aORFs, including those found in *Athila* [12], may be similar to retroviral envelope proteins, we assessed the presence of transmembrane helices (TH) using TMHMM 2.0. A single TH was identified in the middle of the aORF(−) protein of *Athila* 14 (Figure 5B). We also detected an extracellular *N*-terminus and cytoplasmic *C*-terminus, suggesting that the *Athila* 14 aORF(−) protein is related to a type I transmembrane protein [41]. We then checked for the presence of localization signals using DeepLoc 2.1. The predicted location for the *Athila* 14 ORF(−) was the endoplasmic reticulum (probability = 0.6420). Interestingly, we found nuclear localization signals for *Athila* 14 3′aORF(+) and *Tekay* 13-2 5′aORF (RGGKRKL and RKSHRGFLKGLANLLKGKK, respectively), with prediction of nuclear localization probabilities of 0.7991 and 0.7616, respectively (Figure 5C). Additionally, the *Tekay* 13-2 5′aORF protein posed three well-predicted separated a-coils, while other regions of the protein were predicted as disordered (92.5%). The *Tekay* 13-2 5′aORF protein was characterized by a fairly high content of proline (15.9%).

We further investigated the conservation of this 5′aORF and the corresponding protein among the members of *Tekay* group 2 based on LTRs’ similarity. We found 523 *Tekay* group-2-like elements, and 500 (95.6%) of them had 5′aORF, coding for highly similar proteins (Supplementary Figure S9), suggesting high conservation of this 5′aORF within *Tekay* members of the sunflower genome. Then, we searched for similarity of the *Tekay* 13-2 5′aORF protein to the 5′aORFs that were identified among *Tekay* RTEs of *Poaceae* species, including *Z. mays* cv. B73, *Oryza sativa* japonica (*RIRE*3, *RIRE*8A, *RIRE*8B, and *Retrosat*-2), and *Oryza brachyantha* (*FRetro*3) [17,42]. No similarities were observed at the amino acid level. However, similarly to the *Tekay* 13-2 5′aORF protein, all 5′aORFs of other analyzed species exhibited amino acid content biases and had predicted nuclear localization (Supplementary Figure S10).

**Figure 6:**
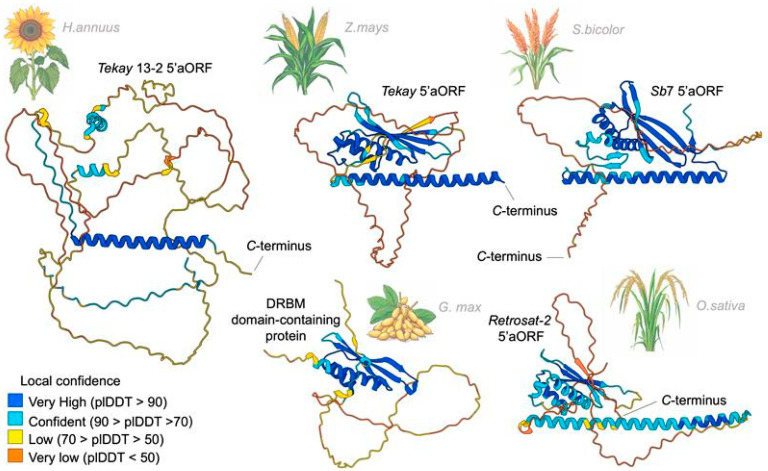
The three-dimensional structures of the *Tekay* 13-2 5′aORF protein and the 5′aORF proteins of *Tekay* elements of other species: *Z. mays* cv. B73 (*Tekay*), *S. bicolor* cv. BTx623 (Sb7), and *O. sativa* japonica (*Retrosat-*2).

Next, we predicted the three-dimensional structures of the proteins encoded by the 5′ and 3′ aORFs using AlphaFold 3. For this purpose, the amino acid sequences were processed on the AlphaFold server and the model with the highest-ranking score was selected (Figure 5C). We found a high-confidence prediction for most secondary structures (predicted local distance difference test (plDDT) > 90). The transmembrane region of *Athila* 14 aORF(−) was folded as an α-coil. *The Athila* 14 aORF(+) protein had predicted structures that included α-coils at the *N*- and *C*-termini and two blocks of three antiparallel β-sheets separated by a region with multiple α-coils. A significant portion of the *Tekay* 13-2 5′aORF protein was disordered, but the NLS (nuclear localization signal) region at the *C*-terminus was folded as an α-coil (plDDT > 70). We then compared the predicted three-dimensional structures of the *Tekay* 13-2 5′aORF protein and the 5′aORF proteins of *Tekay* elements of other species (Figure 6). Interestingly, this analysis revealed similarities in the presence of long disordered regions in all structures, as well as a long α-coil at the *C*-terminus in *Tekay* of *Z. mays* cv. B73 and Sb7 from *Sorghum bicolor* cv. BTx623. The *Retrosat-*2 (*O. sativa* japonica) 5′aORF protein structure had overall lower levels of plDDT, but also included a long α-coil structure, although it was preceded by a long, disordered region at the *C*-terminus. Simultaneously, we noted that the *Poaceae* 5′aORF proteins had similar structures, represented by a complex of α-coils and β-sheets, which were absent in *Tekay* 13-2. To establish the possible role of this protein, we applied the Foldseek search in the 3Di/AA mode. We did not detect significant protein structure similarity for the *Tekay* 13-2 5′aORF protein. However, we found structural similarity of 5′ORF proteins of *Z. mays Tekay* and *S. bicolor Sb*7 (TM-Score: 0.35–0.37; e-value = 3.99 × 10^−2^–2.51 × 10^−1^) with a DRBM-containing protein from *Glycine max* (UniProt ID I1MCB9; Figure 6). Additionally, these results indicated that RTE members of *H. annuus Tekay* group 2 do not possess this feature.

## 3. Discussion

We showed that the *Tekay* elements of group 2 differed from groups 1 and 3 in terms of the distribution of insertions along the chromosome. While the insertions of group 1 and 3 *Tekay* elements were predominantly observed in the pericentromeric regions, the group 2 insertions were randomly distributed over the chromosome arms. *Tekay* elements belong to the Chromovirus clade, which also contains a *CRM* lineage with more frequent insertions near the centromeric regions. *CRM* elements have a unique CR motif at the *C*-terminus of the integrase chromodomain [4]. This feature distinguishes the *CRM* integrase from the integrase of *Tekay* elements that have a type II chromodomain and do not have centromere-biased insertions. According to this, the RTEs of group 2 in our study were typical *Tekay* elements, as they possessed type II chromodomain, their insertions were not biased toward the centromere, and they had 5′aORFs. Consequently, groups 1 and 3 were diverged groups of *Tekay* that had pericentromere-biased insertions and did not carry the 5′aORF while having a type II chromodomain. These results suggested that RTEs with the type II integrase chromodomain can predominantly generate pericentromere insertions. This also indicated that other RTE features (e.g., aORF-encoded proteins) can play an additional role in shaping RTE insertion preferences. 

While we are currently living in the postgenomic era, our knowledge of the functions and structure of proteins encoded by mobile elements in plants is lacking behind the general progress in systems biology. Recent functional studies of eukaryotic and prokaryotic DNA transposons have suggested that mobile elements may encode additional proteins that exhibit different properties. For example, plant DNA transposons of the Vandal family have aORFs that exhibit unique functionalities, such as sequence-specific protection from de novo silencing by the RdDM system [43]. Analysis of bacterial transposons revealed aORFs encoding new classes of RNA-guided DNA endonucleases, such as TnpB (ISDra2 and ISYmu1) proteins [44,45]. These examples demonstrate that, during evolution, DNA transposons may have acquired sequences that encode proteins with different catalytic activities. We found two groups of aORFs proteins with different structural features and localizations in the RTE sequences. The first group of aORFs was located at the 3′ end of the RTE sequence and potentially encoded env-like proteins. It was previously known that plant RTEs may have three additional 3′aORFs encoding env-like proteins [11,12,13]. Here, we also identified *Athila* RTE carrying an ORF for a protein with a transmembrane domain, which is a feature of env-like proteins [12]. Although the functions of env-like proteins are not known, it has been speculated that these proteins may ensure intercellular transport of RTE virus-like particles [12]. Interestingly, human endogenous retroviruses (ERVs) lacking env-encoded ORFs have higher proliferation rates [46]. Thus, the function of aORF-encoded proteins may not be attributed to the increased proliferation rate but can be linked to other RTE properties, such as germline activity and an increased inheritance rate. The second group of aORFs is located at the 5′ end of the RTE sequence and encodes proteins with no structural similarity to env-like proteins. The RTEs of the 5′aORF group have been previously described for *Arabidopsis* and include elements of the *Tekay* clade [10]. Corroborating this, we identified a group of *Tekay* elements that carry 5′aORFs (group 2). The similar 5′aORF sequences were identified in the same position in all elements of this *Tekay* group, but not in groups 1 and 3. The predicted *Tekay* 13-2 5′aOFR protein was quite large (400 aa), well conserved between group-2-related *Tekay* RTEs, and possessed different structural features, including NLS, three well-predicted α-coils, and long disordered regions. Almost 4000 aORFs have been bioinformatically identified in *Ty*1/*Copia* and *Ty*3/*Gypsy* elements across the plant kingdom. These proteins are highly divergent, and most of them lack any conservative domains [47]. Therefore, the putative functions of the non-canonical proteins of RTEs are difficult to predict, and alternative approaches are needed to understand the functions of these proteins. 

Currently, millions of proteins have predicted 3D structures, exhibiting unique opportunities to detect homology between proteins beyond the sequence similarity threshold [48,49]. The structural similarity search assisted by Foldseek [50] significantly accelerated the search process. This approach has recently been used to find structural analogs of viral proteins with unknown functions [51]. Viral proteins have a high divergence rate, resulting in the low efficiency of traditional similarity-based approaches to find homology. A structural similarity search revealed putative functions of multiple viral proteins [51]. To predict the functions of non-canonical proteins in our study, we utilized structural similarity rather than a sequence similarity search between aORF proteins and known proteins with predicted structures in the AlphaFold database [49]. Although we did not detect any hits for sunflower *Tekay* RTEs, we showed that the non-canonical proteins of the *Tekay* family in *Z. mays* and *S. bicolor* were structurally similar to a DRBM-containing protein from *Glycine max*. Plant DRBM proteins possess double-stranded RNA-binding activity and include a wide range of proteins, including Dicer and ADAR family proteins [52]. Interestingly, many plant viruses encode additional proteins, called viral suppressors of RNA silencing (VSRs), which also have double-stranded RNA-binding activity [53]. These proteins help viruses spread and infect plant tissues by counteracting antiviral RNA silencing.

## 4. Materials and Methods

### 4.1. Plant Material

Sunflower seeds of the “ZS” line were provided by the Pustovoit All-Russia Research Institute of Oilseed Crops (Moscow, Russia).

### 4.2. Seed Sterilization and In Vitro Growth Conditions

The seeds were cleaned from the seed coat, washed, and surface-sterilized according to the following steps: (1) washing in 0.02% Tween-20 for 10 min, (2) rinsing in sterile water until the foam disappeared, (3) washing with 70% alcohol for 1 min, (4) surface sterilization in 10% commercial bleach for 3.5 min, and (5) rinsing three times in sterile water for 1 min. Sterilized seeds were then placed on solid MS medium and grown under long-day conditions (16 h light) at 24 °C.

### 4.3. Chemical and Stress Treatment

Ten-day-old plants with developed secondary roots were transferred to MS medium supplemented with a combination of sterile solutions of α-amanitin and zebularine at final concentrations of 2 and 4 μg/mL, respectively. After seven days of growth on toxin-containing media, the plants were transferred at +4 °C for 24 h (cold stress) and then subjected to heat stress at 37 °C for 24 h.

### 4.4. DNA Isolation

Total DNA was isolated using the CTAB protocol for ONT sequencing [54] from whole plants immediately frozen after heat stress and stored at −80 °C. Briefly, 100 mg of whole plant tissue was ground with a mortar and pestle in liquid nitrogen. A total of 0.5 mL of preheated 75 °C CTAB1 buffer containing 6% β-mercaptoethanol and 0.5% polyvinylpyrrolidone was immediately added to the frozen powder. The lysate was transferred to a 1.5 mL tube and incubated at 75 °C for 1 h. After cooling, an equal volume of chloroform was added to each sample, mixed vigorously, and centrifuged at 10,000× *g*. The upper water phase was transferred to a 1.5 mL tube containing 2 volumes of CTAB2 buffer and centrifuged at 100,000× *g*. The obtained pellet was resuspended in 0.2 mL of 1 M NaCl, and DNA was precipitated with an equal isopropanol volume, followed by centrifugation at 10,000× *g*. The pellet was then washed with 70% ethanol and resuspended in nuclease-free water. RNase treatment was performed, followed by isopropanol reprecipitation and 70% ethanol washing. The DNA obtained was resuspended in nuclease-free water for downstream analysis.

### 4.5. eccDNA Isolation

EccDNA fraction enrichment was performed according to a previously described protocol [38]. Briefly, for PS+ samples, linear DNA removal was performed from 1 µg total DNA in a 50 µL reaction by adding 1 μL (10 U) of Plasmid-Safe DNase (LGC Biosearch Technologies, E3101K, Beverly, MA, USA), 2 μL of ATP 25 mM, and 5 μL of 10× Plasmid-Safe buffer. For the PS– samples, all manipulations were performed without enzyme addition. Plasmid-Safe treatment was performed for 72 h at 37 °C with the addition of extra reagents. The reaction was incubated for 72 h with additional reagents (0.1 µL of enzyme, 0.2 µL of ATP, and 0.3 µL of buffer) every 24 h, followed by enzyme inactivation at 72 °C for 30 min. The remaining DNA was precipitated by overnight incubation with 1/10 V 3 M sodium acetate (pH 5.2) and 2.5 V absolute ethanol, followed by centrifugation at 12,000× *g* for 30 min. The obtained PS+ and PS− sample pellets were washed with ice-cold 70% ethanol and dissolved in 10 µL of deionized nuclease-free water. Each sample was then used for eccDNA amplification using random RCA by the addition of 2 µL of phi29 polymerase (Thermo Scientific, EP0091, Waltham, MA, USA), 2 µL of 10× phi29 reaction buffer, 5 µL of 10 mM dNTPs, 1 µL of 500 µM exonuclease-resistant random primer (NpNpNpNpNpSNpSN, where p is the phosphodiester and pS is the phosphorothioate group), and nuclease-free water to a final volume of 20 µL. The reaction mixture was preheated to 95 °C for 5 min, ramped to 30 °C at a 1% ramp rate on a thermocycler, and incubated for 36 h at 30 °C. The enzyme was inactivated by heating the mixture to 65 °C for 10 min. All manipulations upon this step involved two technical replicates. For one repetition of the debranching step, 500 ng of RCA products was treated with T7 endonuclease, 5 µL of 10× reaction buffer and 1 µL of T7 endonuclease I (New England Biolabs, M0302S, Ipswich, MA, USA), in a 50 µL reaction volume. After incubation at 37 °C for 15 min, the reaction was stopped immediately, and the product was purified by adding an equal volume of chloroform. The debranched RCA product was precipitated by adding 1/10 V 3 M sodium acetate (pH 5.2) and absolute ethanol (2.5 V), followed by incubation at −80 °C for 30 min and centrifugation at 12,000× *g* for 30 min. The pellet obtained was dissolved in nuclease-free water. Five hundred nanograms of PS+ RCA+ and PS− RCA+ samples, with or without T7 endonuclease treatment, were used for nanopore sequencing.

### 4.6. eccDNA Validation

Specific primers were designed for inverted PCR to amplify eccDNA junction sites containing one or two LTR sequences (Supplementary Figure S1A). Primer sequences are listed in Table S2. The DNA samples used for validation were PS− RCA− (total DNA without Plasmid-Safe treatment and RCA reaction), PS− RCA+ (sample after RCA reaction without Plasmid-Safe treatment), and PS+ RCA+ (sample after Plasmid-Safe treatment and RCA reaction). 

### 4.7. Library Preparation and Nanopore Sequencing

ONT sequencing library preparation was performed with 500 ng of each DNA sample using Native Barcoding Expansion 1–12 (Oxford Nanopore Technologies (Oxford, UK), Catalog No. EXP-NBD104) and the Ligation Sequencing Kit SQK-LSK110 (Oxford Nanopore Technologies). Sequencing was performed using MinION equipped with an R9.4.1 flow cell. Base-calling was performed using Guppy 6.4.6 (Oxford Nanopore Technologies). 

### 4.8. Transposable Elements’ Annotation

The reference genome HanXRQr2.0-SUNRISE (NCBI RefSeq assembly number GCF_002127325.2) was downloaded from the NCBI database. Due to the highly variable repeatome composition of the genome, annotations of retrotransposons (RTEs) were conducted using a hybrid approach [55], which included two annotation pipelines: (i) domain-based, with the detect_putative_ltr.R script from the DANTE_LTR toolkit v0.3.5.0 (https://github.com/kavonrtep/dante_ltr; with default parameters), followed by the domain-based DANTE tool v0.1.9 (https://github.com/kavonrtep/dante (accessed on 15 August 2024); with default parameters) [56], where the annotation was performed based on the Viridiplantae_v3.0 database (part of REXdb [47]); (ii) structure-based with HiTE, with the following arguments: “--annotate 1 --intact_anno 1” [57]. The HiTE annotation was used as the primary approach and corrected with the DANTE_LTR annotation because of the absence of some RTE annotations, and all annotated TE sequence lineages were determined using TEsorter v.1.4.6 [57,58].

The obtained redundant RTE set was clustered to the family level using MeShClust2 (https://github.com/BioinformaticsToolsmith/MeShClust2 (accessed on 11 September 2024)), with the following arguments: “--id 0.80 --mut-type single --sample 2000 --iterations 30”. Next, clusters with more than one sequence were processed using the homemade Python script “ParseCDHIT_align_get_consensus.py”, which includes alignment with mafft v.7.453 and extraction of consensus or representative sequence. The obtained non-redundant set of RTEs was used for homology-based annotation of fragmented and nested RTEs using RepeatMasker v.4.1.5 (RepeatMasker Open-3.0; http://www.repeatmasker.org (accessed on 21 June 2024)) with rmblast v.2.14.0 as a search engine (https://repeatmasker.org/rmblast/ (accessed on 21 June 2024)) and TRF v.4.09.1 [59]. The analysis was limited to RTEs because the content of the other TE sequences (that is, LINEs, SINEs, and DNA-TEs) in the sunflower genome was negligible [60].

### 4.9. RTE Insertion Time Estimation

The insertion time of each individual intact RTE was estimated using the Kimura 2 parameters method [61]. Calculations were performed using the homemade Python script “Intact_TE_insertion_time.py”. Briefly, LTR pair sequences were aligned using mafft to obtain transition and transversion numbers, and the insertion time was calculated by the following formula: T = K/2r, where T is the insertion time in Mya, K is the genetic distance, and r is the base mutation rate per year, which was taken as r = 1.0 × 10^−8^ [62].

### 4.10. Insertion Detection

Illumina paired-end sequencing data from 12 sunflower accessions (Table S3) [63] were downloaded from the NCBI database. To reduce computational complexity, coverage of the sequencing data was reduced using seqtk v.1.3-r106 (https://github.com/lh3/seqtk, (accessed on 11 June 2024)) with the “sample” option to ×14 genome coverage. The processed files were then aligned to the reference genome using bowtie2 v.2.3.5.1 [64] with the following arguments: “--very-sensitive --mp 13 --rdg 8.5 --rfg 8.5.” The obtained SAM file was converted to the BAM file, sorted, and indexed using SAMtools [65], and PCR duplicates were removed using Picard v.3.1.0 (https://github.com/broadinstitute/picard, (accessed on 13 July 2024)). The insertions were detected using the SPLITREADER pipeline v1.2 [66] with default parameters. TIPs with more than five supporting reads (split or discordant reads) were used for further analysis. The BAM files obtained containing split reads for insertion sites were visualized using JBrowse2 v1.6.5 [67]. The obtained BED files containing insertion sites were processed to find overlaps using the multiIntersectBed program in BEDtools [68].

### 4.11. Circos Plot Drawing and TIPs’ Visualization

To plot TIPs on the chromosome, ggplot2 v.3.5.1 and idiogramFISH v.2.0.13 R packages were used. The positions of the centromeres were identified using the centromere-specific sunflower LINE [69]. Briefly, the sequence of this mobile element was searched against the reference genome using BLASTN v.2.9.0 [70] with default parameters. For the further analysis, hits with more than 70% similarity were taken, the reference genome was split into 100,000 bp windows using “bedtools makewindows”, and hits were counted in each window. Windows with more than 6 hits (identified manually) were assumed as centromeric regions. The pericentromere length was taken as 10% of the chromosome length, and pericentromeric regions of chromosomes were annotated on both sides of the centromere. To plot the distribution of annotated DANTE_LTR and HiTE LTR-RTs, the shinyCircos v.2.0 R package (https://github.com/YaoLab-Bioinfo/shinyCircos-V2.0, (accessed on 1 August 2024)) was used.

### 4.12. Retrotransposon Family Identification

For identification of individual RTE families, the following pipeline was used: first, all annotated long terminal repeats (LTRs) from RTE annotation were extracted from the genome using “bedtools intersect”; then, LTR sequences of interest were searched against the LTRs database using BLASTN and, based on the sequence similarity and alignment length, sequences were characterized as belonging to one family using the 80-80-80 rule [71].

### 4.13. Phylogenetic Analyses and Domain Annotation of RTEs

A multi-FASTA file with 28 RTEs was used to predict the domain structure using TEsorter [62] with the REXdb database [47]. The amino acid sequences of the reverse transcriptase and integrase domains were aligned using the concatenate_domains.py script. The tree file for the resulting alignment file was generated using the iqTree tool [72], with bootstrap (bb) = 1000. The phylogenetic tree was visualized using the iTOL [73] online tool. Domain and open reading frame (ORF) annotations were visualized with gggenes R-studio package (version 0.5.0, https://wilkox.org/gggenes/, accessed on 9 October 2024).

### 4.14. ORF Prediction and Protein Structural Analyses

RTE sequences of families of interest were extracted from genomes using “bedtools intersect”; then, for additional ORFs (adORFs) discovery, the following pipeline was used. First, ORFs were annotated using the “getORFProteins” function with the “minimum_length = 300, remove_nested = True” options from the ORFFinder (https://github.com/Chokyotager/ORFFinder, accessed on 29 June 2024) python package. Next, the protein sequences of manually identified adORFs were searched against the predicted ORFs database using BLASTP, and identified protein sequences were extracted and aligned using mafft. Consensus was extracted using the “dumb_consensus” function of the AlignIO (https://github.com/biopython/biopython, accessed on 9 October 2024) python package. The 5′ aORF RTE sequences were obtained from Steinbauerova et al. [10], particularly those from *Z. mays* cv. B73 [74], *Sb*7 aORF from *S. bicolor* cv. BTx623 [75], and *Retrosat*-2 aORF from *O. sativa* japonica [76]. The software used for the identification of structural properties of the identified proteins included TMHMM 2.0 (https://services.healthtech.dtu.dk/services/TMHMM-2.0/, accessed on 27 October 2024) [77] for transmembrane signals’ discovery, DeepLoc-2.1 (https://services.healthtech.dtu.dk/services/DeepLoc-2.1/, accessed on 27 October 2024) [78] for protein subcellular localization prediction, MobiDB-lite v.3.8.4 (https://github.com/BioComputingUP/MobiDB-lite, accessed on 9 October 2024) [79] for disordered and mobile regions’ prediction, and AlphaFold 3 on the AlphaFold Server (https://alphafoldserver.com, accessed on 27 October 2024) [80] for structural prediction. Structural alignment of proteins was performed via Foldseek (https://search.foldseek.com/, accessed on 27 October 2024) [50] in 3Di/AA mode against the AlphaFold/UniProt50 v4 database. Three-dimensional protein models were visualized via Mol* Viewer (https://molstar.org/viewer/, accessed on 27 October 2024) [81]. 

### 4.15. Code Availability

The code used in this study is available in the GitHub repository (https://github.com/soyboy-hub/SUN_MOBILOME_paper, accessed on 9 June 2024).

## 5. Conclusions

In this study, we identified heterogeneous and phylogenetically different sunflower RTEs that were activated under epigenetic stress. These RTEs exhibited different chromosome insertion distribution patterns. The RTEs belonging to the *Tekay* clade possessed conserved non-canonical proteins found in both sunflower and other plant species, as revealed by protein structure prediction, domain analysis, and conservation studies. This work provided a catalog of diverged and transpositionally active sunflower RTEs with conserved aORFs, paving the way for functional analysis of aORF-encoded proteins in the future. 

## Data Availability

The nanopore data produced for this study are available in the Sequence Read Archive (SRA), NCBI, under Bioproject Accession PRJNA1200253.

## Supplementary Materials

The following supporting information can be downloaded at: https://www.mdpi.com/article/10.3390/ijms252413615/s1.
