# Supplementary material for: Comparative Analysis of Active LTR Retrotransposons in Sunflower (Helianthus annuus L.): From Extrachromosomal Circular DNA Detection to Protein Structure Prediction
Source: Int J Mol Sci. 2024 Dec 19;25(24):13615. doi: 10.3390/ijms252413615 (PMC11728184; doi:10.3390/ijms252413615)
Supplement: Supplementary file 1 [file ijms-25-13615-s001.zip › Supplementary_figures.pdf]

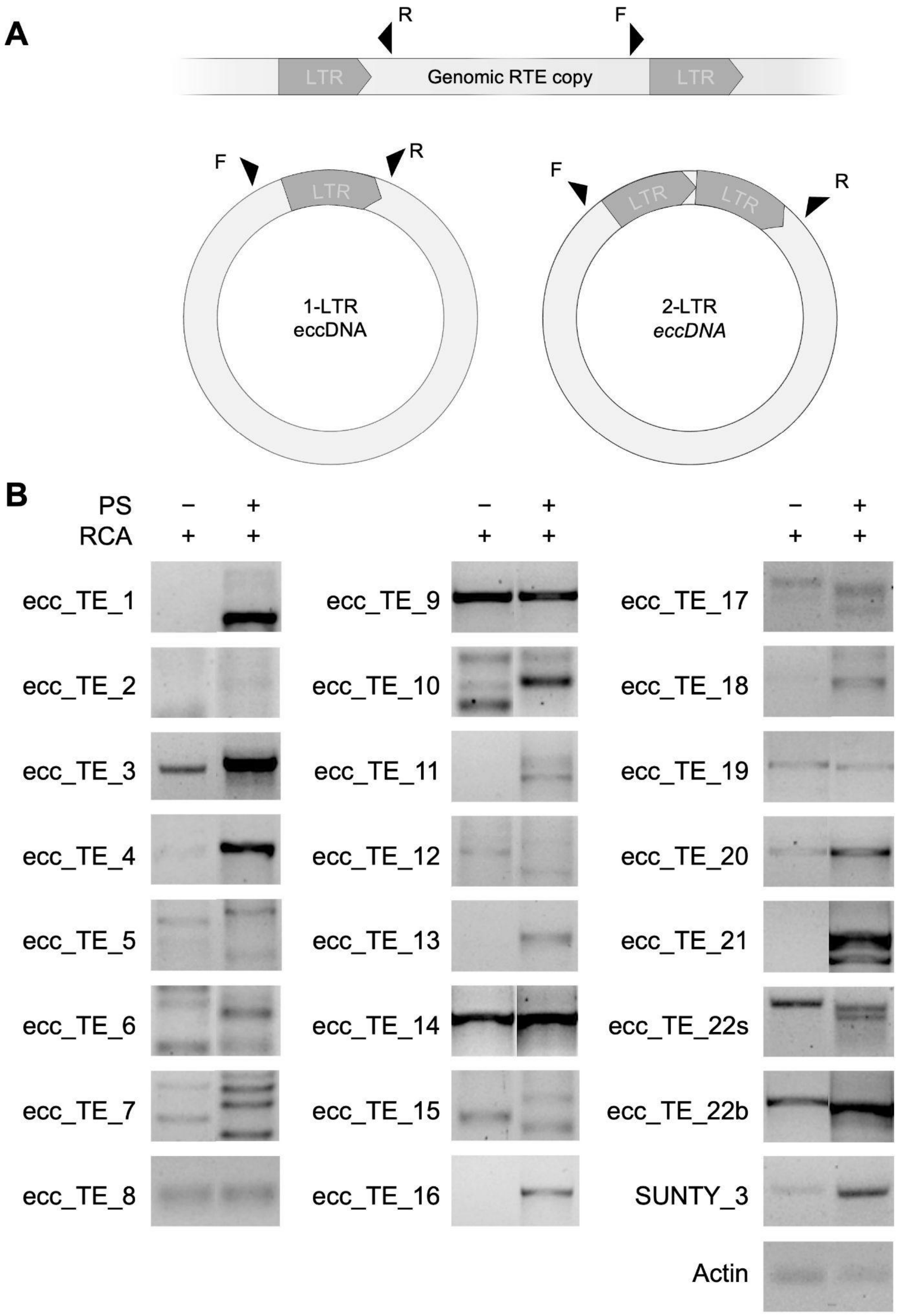

Figure S1. Validation of all active RTEs with primers for inverse PCR: (A) Scheme of primer positions on genomic RTE copy (linear DNA) and RTE eccDNA containing 1 or 2 LTR sequences; (B) Validation of eccDNA in RCA products.

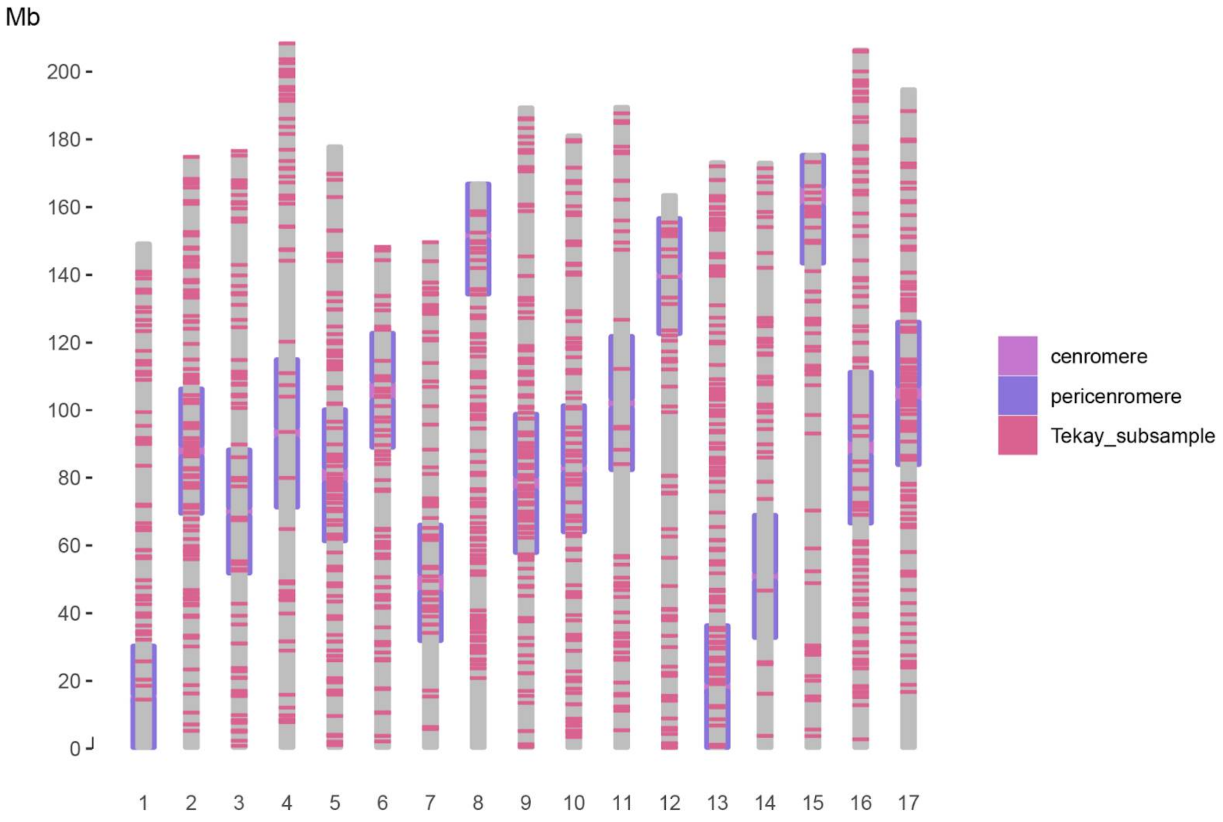

Figure S2. TE insertional polymorphism in *Tekay* subsample group.

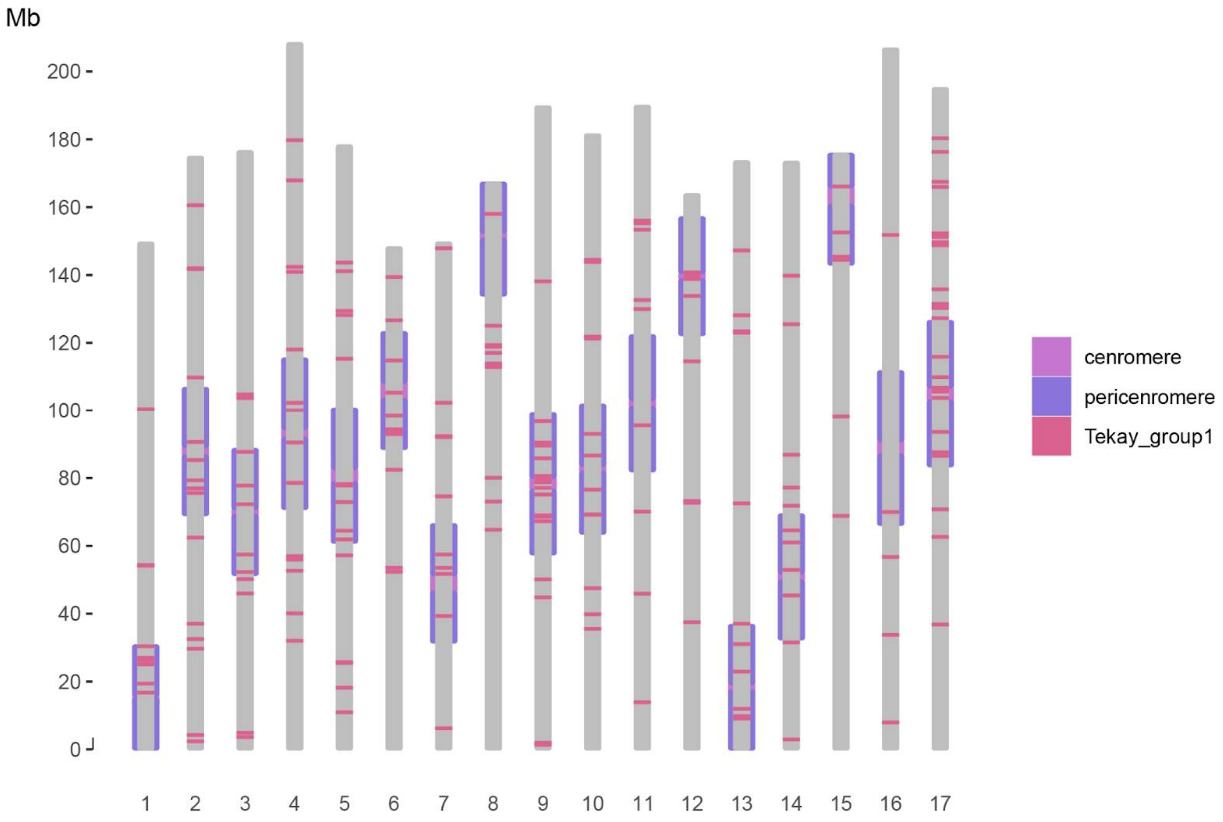

Figure S3. TE insertional polymorphism in *Tekay* group 1.

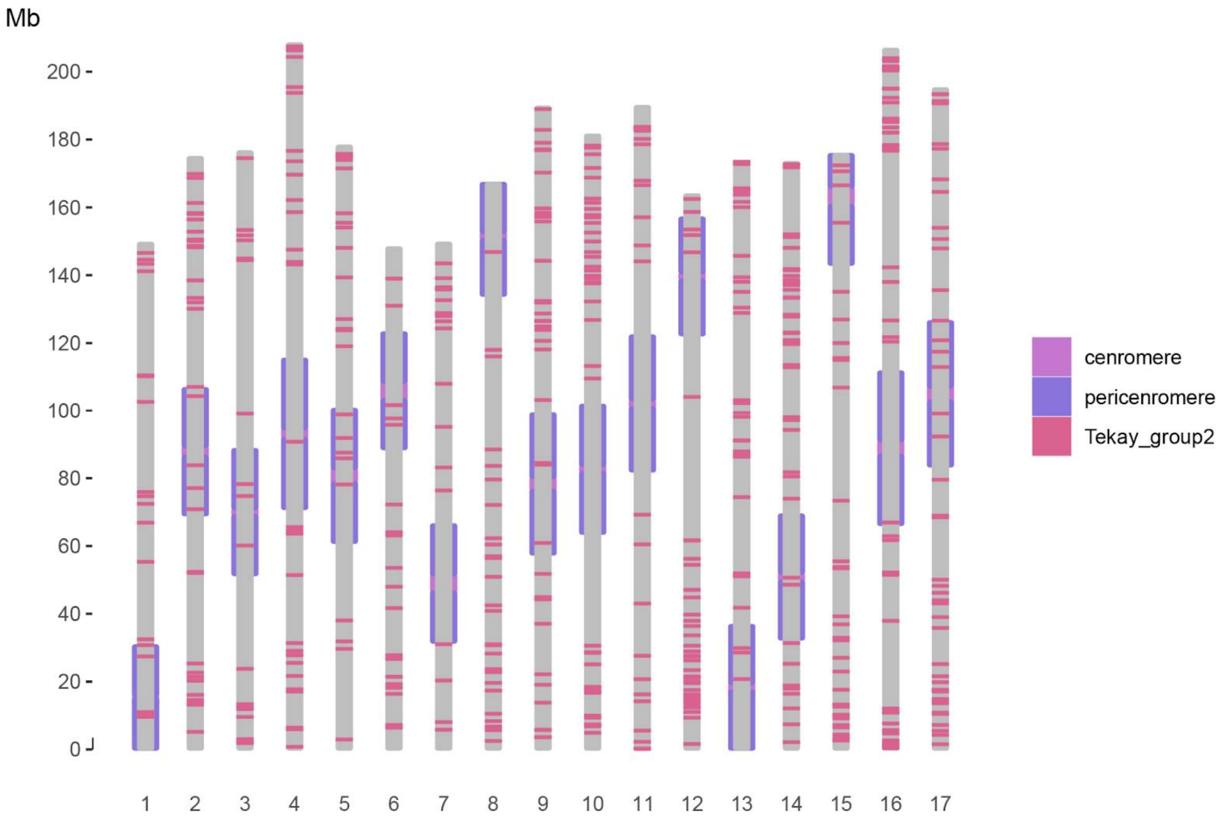

Figure S4. TE insertional polymorphism in *Tekay* group 2.

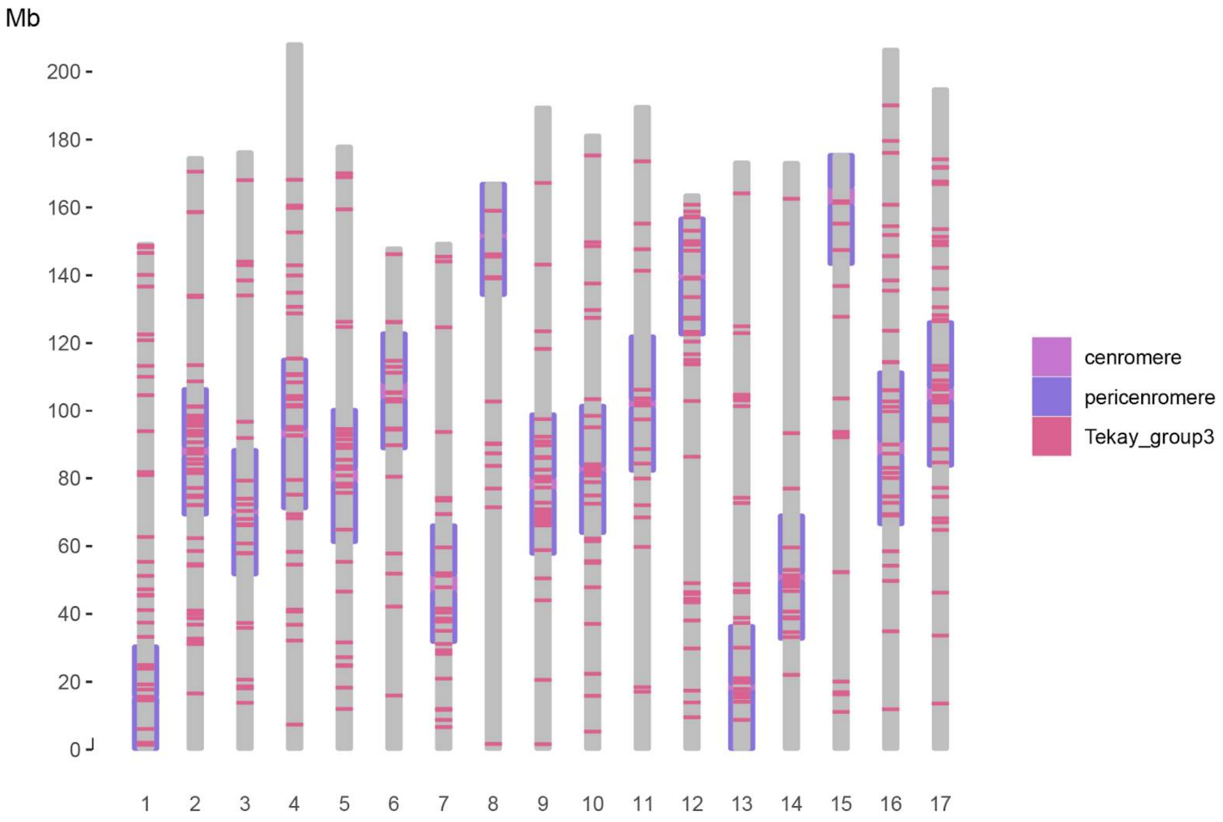

Figure S5. TE insertional polymorphism in *Tekay* group 3.

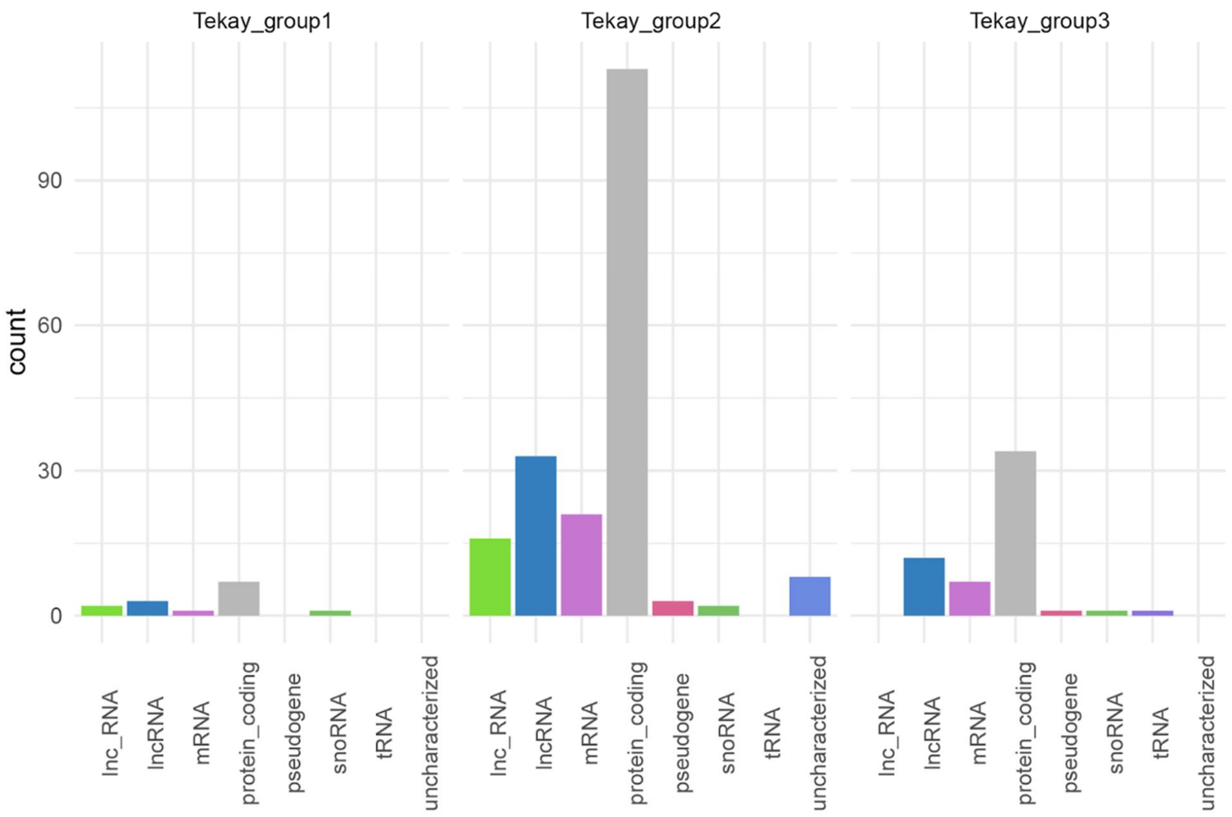

Figure S6. Distribution of *Tekay* gene insertions by gene biotype.

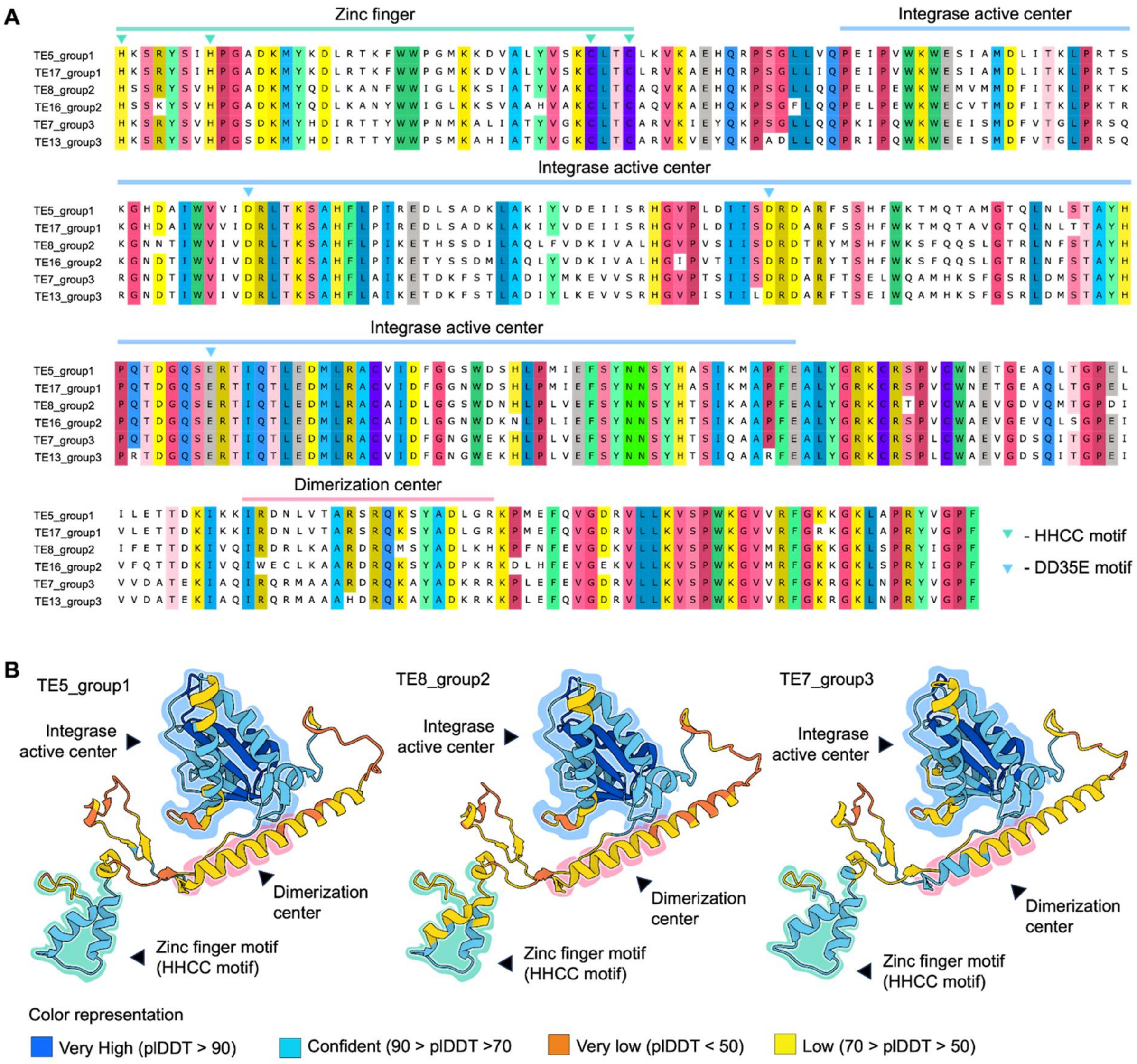

Figure S7. Comparison of integrase amino acid sequences of three Tekay group members with highest TIPs: (**A**) - Multiple alignment of *Tekay* integrase sequences; (**B**) 3D structure models for members of three *Tekay* groups.

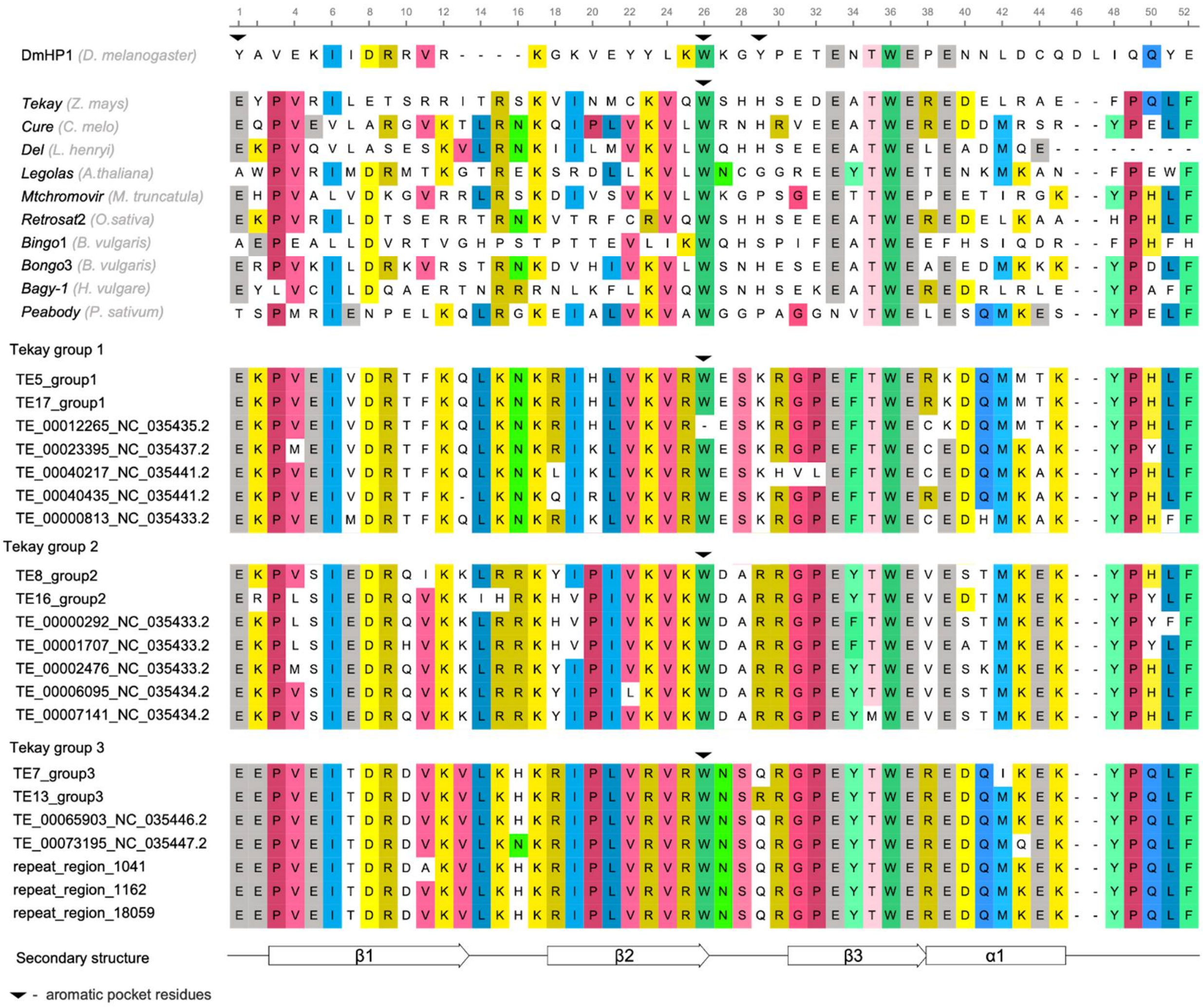

Figure S8. Multiple alignment of "classic" chromodomain HP1 (*D.melanogaster*), chromodomain group II and three *H.annuus Tekay* group chromodomains. Colors applied to >0.25 conservative residues in all sequences used in alignment. Secondary structure for *H.annuus Tekay* RTEs.

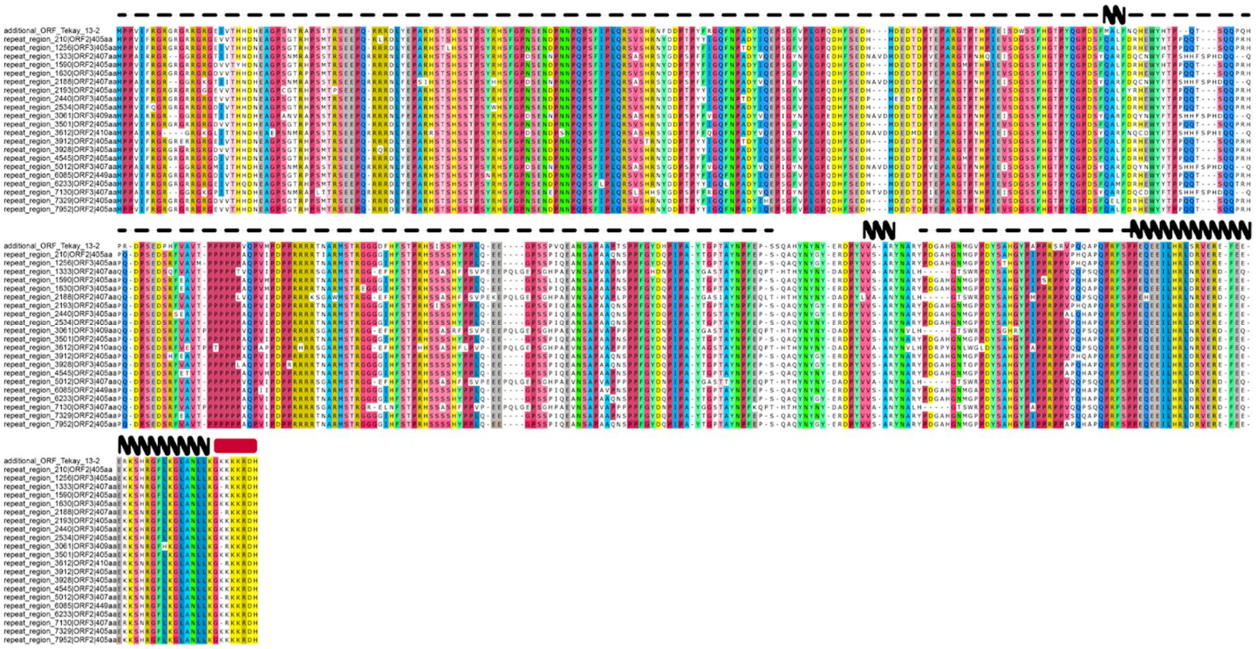

Figure S9. Multiple sequence alignment of Tekay_aORF. Disordered regions, a-coils and nuclear localisation signal are marked by dotted line, coil and red rectangle, respectively.

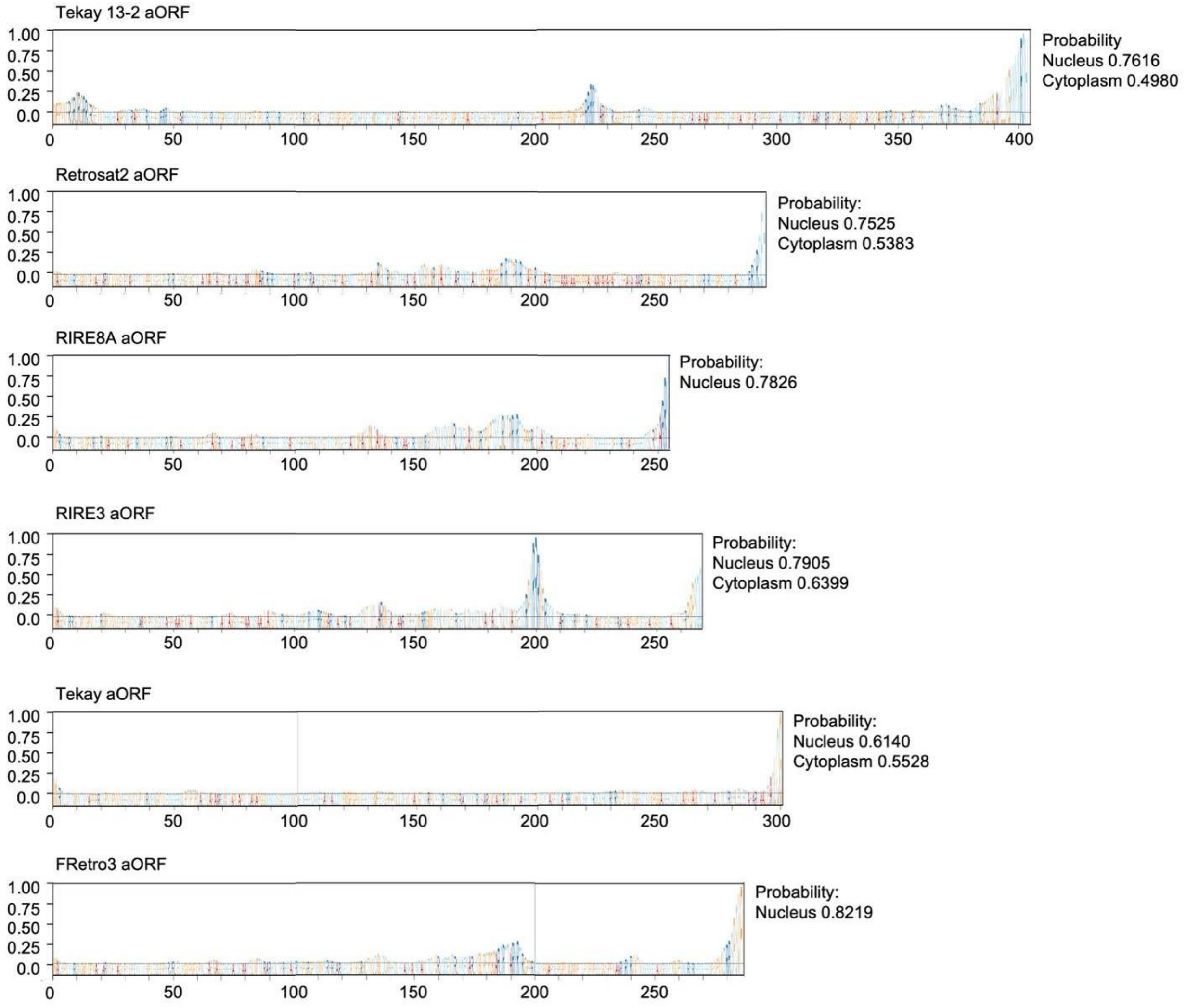

Figure S10. Deeploc 2.1 predicted subcellular localization for 5′ aORFs from H. annuus (Tekay 13-2) and *Tekay* elements of *Poaceae* members.
